# *Vibrio parahaemolyticus* Diarrhea, Chile, 1998 and 2004

**DOI:** 10.3201/eid1101.040762

**Published:** 2005-01

**Authors:** Narjol González-Escalona, Viviana Cachicas, Claudia Acevedo, María L. Rioseco, Juan A. Vergara, Felipe Cabello, Jaime Romero, Romilio T. Espejo

**Affiliations:** * University of Chile, Santiago, Chile; † Hospital Regional de Puerto Montt, Puerto Montt, Chile; ‡ New York Medical College, Valhalla, New York, USA

**Keywords:** Vibrio, parahaemolyticus, pandemic clone, Chile, AP-PCR

## Abstract

Analysis of clinical isolates of *Vibrio parahaemolyticus* from outbreaks in Chile in the cities of Puerto Montt in 2004 and in Antofagasta in 1998 indicated that 23 of 24 isolates from Puerto Montt and 19 of 20 from Antofagasta belonged to the pandemic clonal complex that emerged in Southeast Asia in 1996.

*Vibrio parahaemolyticus* infections that caused most seafoodborne gastroenteritis were associated with multiple diverse serotypes until 1996. Since then, however, most cases have been caused by the **O3:K6 serotype** (*1*). Molecular studies with pulsed-field gel electrophoresis (PFGE) (*2*), arbitrarily primed polymerase chain reaction (AP-PCR) (*3*,*4*), and multilocus sequence typing (MLST) (*5*) have shown that these pandemic strains are clonally related. Most isolates of this pandemic complex exhibit a unique sequence within the *toxRS* operon (*toxRS* */new*) (*4*) and possess a unique open reading frame, *orf8* (*6*), which corresponds to an associated filamentous phage. Other common properties of pandemic strains are the presence of the structural *tdh* gene and the absence of *trh* and urease gene (*7*). By using the criteria stated above, strains of the pandemic clone have been identified as dominant isolates from clinical cases of diarrhea reported in various Southeast Asian countries, including India, Japan, Thailand, Bangladesh, Taiwan, and Vietnam, as well as from some cases in the United States (*1*,*2*,*4*,*8*,*9*) but not in the Southern Hemisphere. Most epidemic isolates initially identified were classified within serovar O3:K6, however, more recently, isolates classified in serovars other than O3:K6 have been identified as also forming part of the pandemic clone (*4*,*8*,*10*,*11*).

From 1992 to 1997, the Institute of Public Health (ISP) reference laboratory in Chile received 30 isolates from regional hospital laboratories for identity confirmation. However, an outbreak that occurred primarily in the northern city of Antofagasta (23°39′S 70°24′W) from November 1997 to March 1998 caused this number to increase to >300 isolates (*12*). A second outbreak affecting approximately 1,500 persons (Unidad de Epidemiología, Servicio de Salud Llanquihue-Chiloé-Palena, Chile, pers. comm.) occurred from January to March 2004, mainly in Puerto Montt (41°29′S 72°24′W), a region of usually cold coastal water. Apart from its public health impact, this last outbreak had important economic and social repercussions because this region is one of the main shellfish-producing areas in Chile. The presence of *tdh*-positive *V. parahaemolyticus* was confirmed in random shellfish samples, and the extraction of seafood had to be suspended during the season of high demand.

## The Study

To investigate the identity and relationship of the *V. parahaemolyticus* strains causing these two outbreaks to the pandemic strains, we determined their phenotypic and genotypic properties. *V. parahaemolyticus* isolates from Puerto Montt were obtained from rectal swabs from 24 patients 6 to 69 years of age with acute diarrhea. The 20 clinical isolates from Antofagasta were provided by the National Institute of Public Health of Chile. Eleven previously well-characterized strains of the pandemic complex, isolated in Southeast Asia, including strain RIMD2210633 (VpKX), whose genome has been sequenced (*13*), were included for comparison. VpKX was directly obtained from the culture collection. The other bacterial strains of the pandemic group were provided by Mitsuaki Nishibuchi, Center for Southeast Asian Studies, Kyoto University. They are KXV225, VP2, VP47, VP81, 97LVP2, JKY-VP6, AN-5034, AN-8373, OP-424, KXV737. Three other *V. parahaemolyticus* nonpandemic strains, ATCC17802T (VpD), RIMD 2210856 (VpAQ), 2210086 (VpI), and the *V. alginolyticus* strain ATCC17749 (Va) were directly obtained from the indicated culture collections. The identification of the isolates used in this study was confirmed by API-20E for enterobacteria (bioMérieux, Inc., Hazelwood, MO), according to the manufacturer’s instructions. The O and K antigens of the *V. parahaemolyticus* strains were determined by slide agglutination with rabbit antisera obtained from Seiken (Denka Seiken. Co. Ltd. Tokyo, Japan), as described by the supplier.

For PCR, bacterial DNA was extracted from overnight cultures in Luria Bertani broth (supplemented with 1% NaCl) by using the Wizard Genomic DNA Purification kit (Promega, Madison, WI). DNA concentration was assessed by the intensity of the DNA band after agarose gel electrophoresis and staining with ethidium bromide. Known amounts of λ-DNA were used as a standard. PCR assays were performed by using approximately 10 ng per reaction tube, except for AP-PCR, in which 25 ng were employed. Amplifications of the different markers were performed as previously described: *tdh* and *trh* (*7*), *orf8* (*11*), *toxRS/new* (*4*), and AP-PCR (*11*). The AP-PCR patterns were recorded and analyzed with GelCompar II (Applied Maths, Sint-Martens-Latern, Belgium). Genetic distance was calculated on the basis of the number of shared bands between isolates, and similarity matrices were calculated by using the Dice coefficient (*14*). Clustering was achieved by using the unweighted pair group method with arithmetic mean (UPGMA). Kanagawa and urease tests were performed as described by the U.S. Food and Drug Administration/Bacterial Analysis Manual (*15*).

Twenty *V. parahaemolyticus* isolates obtained from the outbreak in Antofagasta, Chile, in 1998 and 24 from the outbreak of Puerto Montt, Chile, in 2004 were characterized. All of the isolates, excluding 1 from Antofagasta and 6 from Puerto Montt, tested positive for every property of the pandemic clonal complex under analysis (Table). Only 1 isolate from Antofagasta (ATC 230) and 1 from Puerto Montt (PMC 59) differed from the pandemic group in more than 1 property. Five isolates from Puerto Montt diverged from the main group in only 1 of the tested properties; 4 were *toxRS/new* negative, and 1 was serovar O4:K12. The isolates were also analyzed by their AP-PCR patterns. For this analysis, the patterns for 11 South East Asia isolates of the pandemic clone were initially obtained. All of these isolates could be clustered into only 2 groups when either primer P1 or P3 was employed. The patterns obtained with the isolates from Chile were then compared with those of 3 isolates from Southeast Asia, representing the 2 AP-PCR patterns observed within this last group. Three nonpandemic *V. parahaemolyticus* strains and 1 *V. alginolyticus* strain were also included. Every isolate from Chile, except ATC230, clustered with the pandemic isolates when using primer P1 (*11*) for PCR (Figure A). A similar result was observed with primer P3 (Figure B), although in this case PMC59, the isolate that differed from those in the clonal complex in more than 1 property (Table) clustered with ATC230.

**Table:** Properties of *Vibrio parahaemolyticus* strains isolated from outbreaks in Antofagasta, 1998, and Puerto Montt, 2004, in Chile and of strains from culture collections

Strain	Serotype	*tdh*	*trh*	Urease	*orf8*	Ka	*toxRS/new*
Southeast Asia							
VpD	O1:K1			+	–	–	–
VpI	O4:K12	+	–	–	–	–	–
VpAQ	O3:K6	+	+	+	–	–	–
VpKX	O3:K6	+	–	–	+	+	+
Chile, Antofagasta, 1998							
ATC: 208, 210, 211, 213, 214, 216, 217, 218-227, 231, 232	O3:K6	+	–	–	+	+	+
ATC 230	O1.K56	+	+	+	–	+	–
Chile, Puerto Montt, 2004							
PMC:33, 34, 36, 41, 42, 47-49, 52, 53, 55, 57, 58, 60, 62, 65, 69, 72	O3:K6	+	–	–	+	+	+
PMC-46	O4:K12	+	–	–	+	+	+
PMC-61, 66, 67, 68	O3:K6	+	–	–	+	+	–
PMC-59	O4:K12	+	+	+	+	+	–

**Figure:**
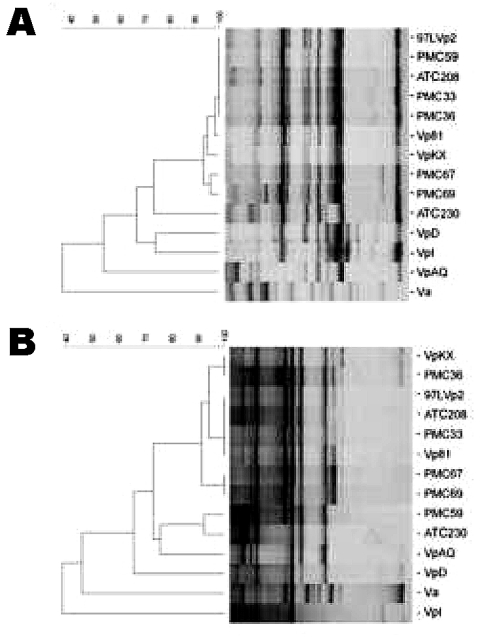
Representative arbitrarily primed polymerase chain reaction (AP-PCR) patterns for *Vibrio parahaemolyticus* DNA from strains of the outbreaks in Chile and Southeast Asia and dendrogram illustrating the clustering of the patterns by similarity. The percentage of similarity is shown above the dendrogram. A and B are AP-PCR with primers P1 and P3, respectively. The corresponding isolates are indicated on the right. PMC and ATC correspond to isolates from the outbreaks in Puerto Montt and Antofagasta, respectively. VpD, VpKX, VpAQ and VpI correspond to culture collection strains ATCC17802T, RIMD2210633, RIMD 2210856, and RIMD 2210086, respectively. 97LVp2 and Vp81 correspond to strains obtained from Mitsuaki Nishibuchi of the Center for Southeast Asian Studies, Kyoto University.

## Conclusions

Our results show that the spread of the pandemic clonal complex reached the Southern Hemisphere as early as 1998, only 2 years after the strain abruptly appeared in Calcutta, India, in 1996 (*3*). The diversity observed among the isolates obtained in Puerto Montt did not appear among those from Antofagasta. Five isolates differed in only 1 property, 1 was from a different serovar, and 4 lacked the *toxRS/new* sequence. These findings raise the question of whether this diversity was originally present in the introduced pandemic strain or whether it was locally generated. The serovar O4:K12 observed in 1 of these isolates (PMC46) has been previously observed among isolates from southern Thailand (*11*). How and when the pandemic strain arrived to these remote regions in the Southern Hemisphere, and why it caused the outbreaks during those years, remains a matter for speculation. The outbreak in Puerto Montt was likely triggered by higher than normal temperatures during the summer months in this region, which is normally cool in all seasons and has an average daily superficial water temperature<16°C year round (Dirección Meteorológica de Chile, pers. comm.).

## References

[1] Bag PK, Nandi S, Bhadra RK, Ramamurthy T, Bhattacharya SK, Nishibuchi M, Clonal diversity among recently emerged strains of *Vibrio parahaemolyticus* O3:K6 associated with pandemic spread. J Clin Microbiol. 1999;37:2354–7. 10364615 10.1128/jcm.37.7.2354-2357.1999 PMC85163

[2] Wong HC, Liu SH, Wang TK, Lee CL, Chiou CS, Liu DP, Characteristics of *Vibrio parahaemolyticus* O3:K6 from Asia. Appl Environ Microbiol. 2000;66:3981–6. 10.1128/AEM.66.9.3981-3986.2000 10966418 PMC92248

[3] Okuda J, Ishibashi M, Hayakawa E, Nishino T, Takeda Y, Mukhopadhyay AK, Emergence of a unique O3:K6 clone of *Vibrio parahaemolyticus* in Calcutta, India, and isolation of strains from the same clonal group from Southeast Asian travelers arriving in Japan. J Clin Microbiol. 1997;35:3150–5. 9399511 10.1128/jcm.35.12.3150-3155.1997 PMC230139

[4] Matsumoto C, Okuda J, Ishibashi M, Iwanaga M, Garg P, Rammamurthy T, Pandemic spread of an O3:K6 clone of *Vibrio parahaemolyticus* and emergence of related strains evidenced by arbitrarily primed PCR and toxRS sequence analyses. J Clin Microbiol. 2000;38:578–85. 10655349 10.1128/jcm.38.2.578-585.2000 PMC86152

[5] Chowdhury NR, Stine OC, Morris JG, Nair GB. Assessment of evolution of pandemic *Vibrio parahaemolyticus* by multilocus sequence typing. J Clin Microbiol. 2004;42:1280–2. 10.1128/JCM.42.3.1280-1282.2004 15004094 PMC356825

[6] Nasu H, Iida T, Sugahara T, Yamaichi Y, Park KS, Yokoyama K, A filamentous phage associated with recent pandemic *Vibrio parahaemolyticus* O3:K6 strains. J Clin Microbiol. 2000;38:2156–61. 10834969 10.1128/jcm.38.6.2156-2161.2000 PMC86752

[7] Suthienkul O, Ishibashi M, Iida T, Nettip N, Supavej S, Eampokalap B, Urease production correlates with possession of the trh gene in *Vibrio parahaemolyticus* strains isolated in Thailand. J Infect Dis. 1995;172:1405–8. 10.1093/infdis/172.5.1405 7594689

[8] Bhuiyan NA, Ansaruzzaman M, Kamruzzaman M, Alam K, Chowdhury NR, Nishibuchi M, Prevalence of the pandemic genotype of *Vibrio parahaemolyticus* in Dhaka, Bangladesh, and significance of its distribution across different serotypes. J Clin Microbiol. 2002;40:284–6. 10.1128/JCM.40.1.284-286.2002 11773134 PMC120132

[9] Chowdhury NR, Chakraborty S, Ramamurthy T, Nishibuchi M, Yamasaki S, Takeda Y, Molecular evidence of clonal *Vibrio parahaemolyticus* pandemic strains. Emerg Infect Dis. 2000;6:631–6. 10.3201/eid0606.000612 11076722 PMC2640929

[10] Chowdhury NR, Chakraborty S, Eampokalap B, Chaicumpa W, Chongsa-Nguan M, Moolasart P, Clonal dissemination of *Vibrio parahaemolyticus* displaying similar DNA fingerprint but belonging to two different serovars (O3:K6 and O4:K68) in Thailand and India. Epidemiol Infect. 2000;125:17–25. 10.1017/S0950268899004070 11057955 PMC2869565

[11] Laohaprertthisan V, Chowdhury A, Kongmuang U, Kalnauwakul S, Ishibashi M, Matsumoto C, Prevalence and serodiversity of the pandemic clone among the clinical strains of *Vibrio parahaemolyticus* isolated in southern Thailand. Epidemiol Infect. 2003;130:395–406. 12825723 PMC2869975

[12] Cordova JL, Astorga J, Silva W, Riquelme C. Characterization by PCR of *Vibrio parahaemolyticus* isolates collected during the 1997–1998 Chilean outbreak. Biol Res. 2002;35:433–40. 10.4067/S0716-97602002000300017 12462995

[13] Makino K, Oshima K, Kurokawa K, Yokoyama K, Uda T, Tagomori K, Genome sequence of *Vibrio parahaemolyticus*: a pathogenic mechanism distinct from that of V cholerae. Lancet. 2003;361:743–9. 10.1016/S0140-6736(03)12659-1 12620739

[14] Nei M, Li WH. Mathematical model for studying genetic variation in terms of restriction endonucleases. Proc Natl Acad Sci U S A. 1979;76:5269–73. 10.1073/pnas.76.10.5269 291943 PMC413122

[15] Kaysner CA, DePaola A. *Vibrio cholerae, V. parahaemolyticus, V. vulnificus*, and other *Vibrio* spp. Bacteriological analytical manual, 8th ed., rev A. Washington: Food and Drug Administration; 1998. Chap. 9.

